# Paracingulin controls the junctional accumulation of nonmuscle myosin-2 in endothelial cells in vivo

**DOI:** 10.17912/micropub.biology.001602

**Published:** 2025-06-24

**Authors:** Florian Rouaud, Isabelle Mean, Lionel Jond, Sandra Citi

**Affiliations:** 1 Department of Molecular and Cellular Biology, University of Geneva

## Abstract

The localization of nonmuscle myosin-2 (NM2) isoforms in endothelial cells and the mechanisms that regulate their localizations are poorly understood. Here we show that NM2A and NM2B are localized at junctions of mouse aortic endothelial cells in vivo, whereas only NM2B is detectable at junctions of cultured bEnd.3 endothelial cells. In both models, the knockout of the junctional protein paracingulin results in the loss of the junctional localization of NM2s. These results demonstrate the physiological relevance of our previous in vitro observations on epithelial cells, provide a mechanism for the localization of NM2s at endothelial junctions, and raise new questions for future studies.

**Figure 1. Paracingulin recruits nonmuscle myosins to endothelial junctions in vivo and in vitro:**
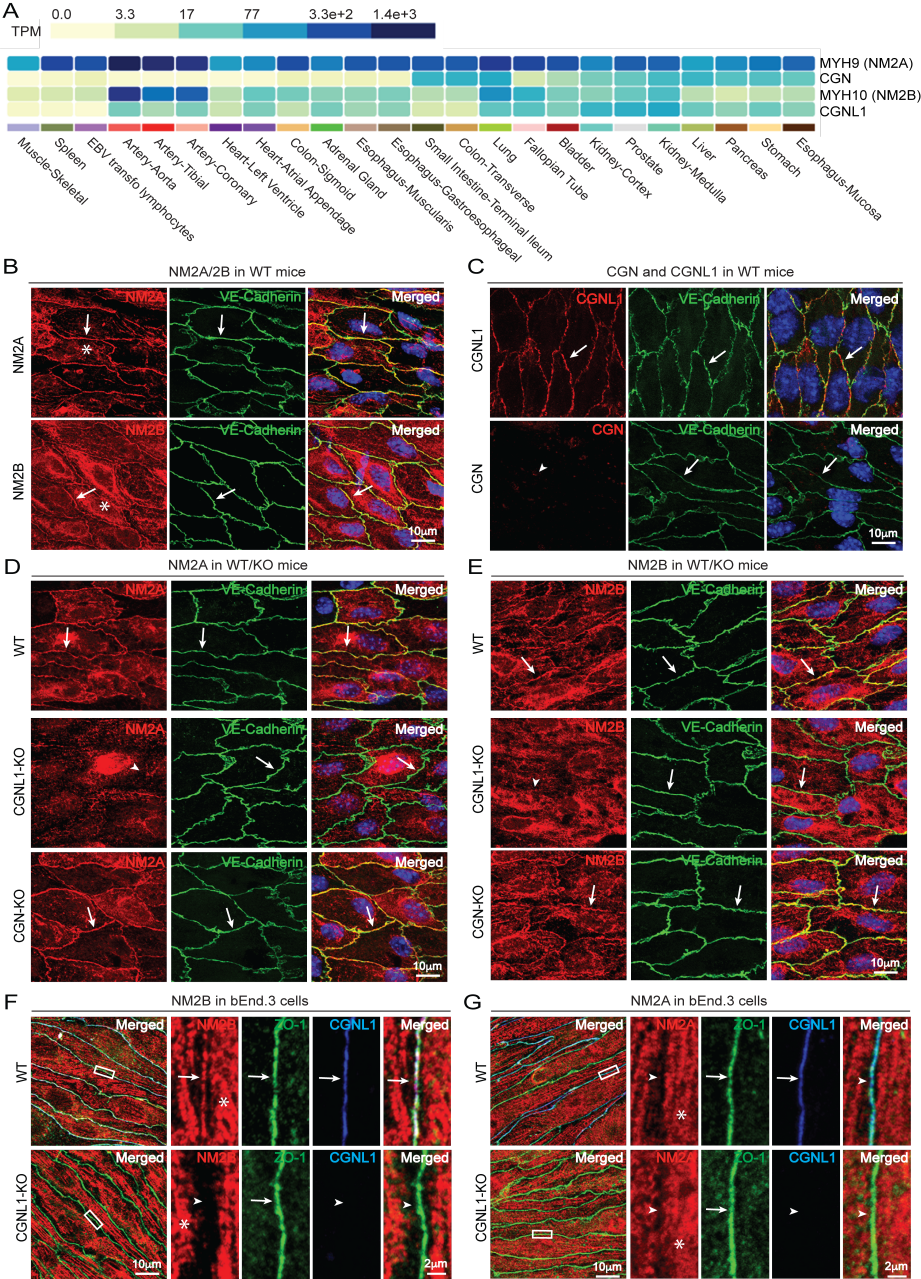
(A) Analysis of datasets from GTexPortal (https://www.gtexportal.org/home /multiGeneQueryPage/CGN,CGNL1,MYH9,MYH10 accessed on 19 February 2025). TPM refers to Transcript Per Million. Color code (light green to dark blue) correlates with transcript levels (from light green = lowest to dark blue = highest). (B-C) Immunofluorescence (IF) microscopy analysis of the localization of endogenous NM2A, NM2B (B), CGNL1 and CGN (C) in endothelial cells of aorta in WT mice. VE-Cadherin labeling is used as junctional reference marker. Arrows and arrowheads show normal and decreased/undetected junctional labeling, respectively. Asterisks indicate cytoplasmic labeling for NM2s. Scale bars = 10 μm. (D-E) IF microscopy analysis of junctional labeling of NM2A (C) and NM2B (D) in WT, CGNL1-KO and CGN-KO mice. Arrows and arrowheads show normal and decreased/undetected junctional labeling, respectively. Scale bars = 10 μm. (F-G) IF microscopy analysis of junctional labeling of NM2B (F) and NM2A (G) in WT and CGNL1-KO bEnd.3 cells. ZO-1 labeling is used as a junctional reference marker. Magnified images on the right highlight the junctional region, labelled by ZO-1 antibodies. Arrows and arrowheads show normal and decreased/undetected junctional labeling respectively. Asterisks indicate cytoplasmic labeling. Scale bars: 10 μm (left images); 2 μm (magnifications on the right).

## Description

The actomyosin cytoskeleton is fundamentally important in cell and tissue physiology, since it controls multiple activities and functions, such as cell division, cell migration, morphogenesis, cell shape and permeability barrier function of epithelia and endothelia. The main components of the actomyosin cytoskeleton are actin and myosin, and three paralogs of NM2s are expressed in mammalian nonmuscle cells, e.g. nonmuscle myosin 2A (NM2A), nonmuscle myosin 2B (NM2B) and nonmuscle myosin 2C (NM2C). These isoforms are coded by the *MYH9, MYH10 and MYH14* genes, respectively, and play unique and overlapping functions in developing and adult tissues (Conti, et al, 2004; Smutny et al, 2010; Ma et al, 2010; Ma et al, 2020)(reviewed in Conti and Adelstein 2008; Shutova and Svitkina, 2018; Sellers and Heissler 2019; Quintanilla et al, 2023). In nonmuscle and epithelial cells NM2 filaments are distributed in the cytoplasm and along the cellular cortex and stress fibers, but can also be concentrated at specific sites, for example at cell-cell and cell-substratum junctions (Drenckhahn et al 1983; Citi et al 1991; Maupin et al 1994), where tensile forces are applied and generated (Charras and Yap, 2018). Nonmuscle myosin was detected in aortic endothelium by immunofluorescence (Borrione et al, 1990), and further analysis showed that only NM2A and NM2B, but not NM2C, are expressed in endothelial cells of the developing mouse (Yoon et al, 2019; Ma et al, 2020). However, to our knowledge the junctional localization of specific NM2 isoforms and the mechanisms that control their localization in endothelial cells in vivo have not been investigated (Bazzoni and Dejana, 2004; Castro-Dias et al, 2019). Conversely, in cultured endothelial cells it was shown that NM2A was redistributed from junctions to stress fibers upon depletion of either ZO-1 or VE-cadherin (Tornavaca et al 2015). However, we previously demonstrated that ZO-1 does not directly bind to NM2s, but it functions by recruiting to junctions cingulin (CGN) and paracingulin (CGNL1), which in turn directly bind to NM2B and NM2A through coiled-coil rod mediated interactions to promote NM2 accumulation at apical cell-cell junctions (Rouaud et al 2023). Specifically, rescue and overexpression experiments in the background of CGN-KO and CGNL1-KO cultured epithelial cells showed that cingulin promotes the junctional accumulation of NM2B, whereas paracingulin promotes the junctional accumulation of both NM2A and NM2B, whereas neither cingulin nor paracingulin affect the localization of NM2C (Rouaud et al 2023). However, since those experiments were carried out in cultured cells, the physiological relevance of our observations remained to be established. To address this question, here we examined the localization of NM2A and NM2B in endothelial cells of intact aortas in WT mice and in mice KO for either cingulin or paracingulin. In addition, we explored the localization of NM2A and NM2B in cultured bEnd.3 endothelial cells, and the effect of KO of paracingulin.

First we analyzed the mRNA expression profiles of NM2A, NM2B, cingulin and paracingulin across tissue types, and examined their localization in endothelial cells using specific antibodies that were previously validated in knock-out and knock-down cells and tissues (Rouaud et al 2023; Flinois et al, 2024). Analysis of GTEx Portal datasets shows that both NM2A and NM2B mRNAs are detectable in arteries, and that NM2A mRNA is expressed at high levels in different types of epithelial tissues ( Fig. 1A ). NM2C was not analyzed because previous studies showed that it is not expressed in mouse endothelium (Ma et al, 2020). To examine the localization of NM2 isoforms in endothelial cells in vivo, we carried out immunofluorescence microscopy of aorta using a whole-mount protocol, where endothelial cells are examined as an intact monolayer in situ, without detaching them from the arterial wall. VE-cadherin (Lampugnani et al, 1992) was used as a reference marker for endothelial junctions. In WT aorta labeling for both NM2A (arrow in Fig. 1B, top panel) and NM2B (arrow in Fig. 1B, bottom panel) was accumulated at VE-cadherin positive endothelial cell-cell junctions (arrows in Fig. 1B ). Labeling for NM2A and NM2B and was also localized throughout the cortex/cytoplasm, presumably corresponding to cytoplasmic filaments and stress fibers (asterisks in Fig 1B). Concerning cingulin and paracingulin, analysis of GTEx portal datasets showed that the expression of both *CGN* and *CGNL1* genes is high in epithelial tissues and organs, but only paracingulin mRNA is detected at high levels in arteries ( Fig. 1A ). In agreement, when we analyzed the expression of cingulin and paracingulin by immunofluorescence of aortic endothelial cells, CGNL1 labeling showed a sharp accumulation at junctions and was largely co-localized with VE-cadherin (arrows, Fig. 1C ), whereas junctional cingulin was undetectable (arrowhead, Fig. 1C ).

Next, we compared the localization of NM2A ( Fig. 1D ) and NM2B ( Fig. 1E ) in aortic endothelium from either WT (top panel, Fig. 1D ), or CGNL1-KO (middle panel, Fig. 1D ) or CGN-KO mice (bottom panel, Fig. 1D ). Strikingly, the junctional localization of both NM2A and NM2B was lost in CGNL1-KO aortic endothelial cells (arrowheads in Fig. 1D and 1E, middle panels). In contrast, both WT and CGN-KO aortic endothelial cells showed a normal junctional localization of both NM2A and NM2B (arrows, Fig. 1D and Fig. 1E, top and bottom panels), indicating that cingulin is not involved in NM2 localization in aortic endothelial cells, because it is not detectably expressed. Interestingly, the localization of VE-cadherin was very similar in WT and KO mice (arrows in green channel, Fig. 1D- E), indicating that the integrity of endothelial junctions is not grossly affected by the loss of either cingulin or paracingulin. This is consistent with the observation that mice KO for either cingulin or paracingulin are viable, with no obvious vascular defect under baseline conditions (Guillemot et al, 2012; Flinois et al, 2024). To confirm these results in a different type of endothelial cell, we generated a knockout of CGNL1 in cultured bEnd.3 cells using CRISPR-Cas9 technology, resulting in loss of paracingulin expression by immunoblotting (Extended Data Figure) and immunofluorescence microscopy ( Fig. 1F- G, WT in top panels and CGNL1-KO in bottom panels). Interestingly, in WT bEnd3 cells NM2B was localized both at ZO-1-labelled cell-cell junctions and in the cytoplasm/cortex (arrow and asterisk, Fig. 1F ), whereas NM2A was not detectable at junctions, but was accumulated in the cytoplasmic/cortical area (arrowhead and asterisk, Fig. 1G ). Importantly, no junctional labeling for NM2B was detected in CGNL1-KO bEnd.3 cells (arrowhead in Fig. 1F, bottom panel). The labeling for both NM2A (arrowheads, Fig. 1G ) and ZO-1 (arrows, Fig. 1F- G) was similar in WT and CGNL1-KO bEnd.3 cells. In summary, the KO of paracingulin results in the loss of junctional NM2A and NM2B in aortic endothelial cells in vivo, and the loss of junctional NM2B in bEnd.3 cells in vitro, with no apparent effect on the integrity of endothelial junctions.

Our results advance our understanding of the subcellular distribution of NM2s in intact endothelia in vivo, by describing the localizations of NM2A and NM2B isoforms in the aorta. Moreover, they provide to our knowledge the first reported mechanism for the localization of NM2A and NM2B at endothelial junctions in vivo. Previous experiments in vitro using cultured human dermal microvascular endothelial cells showed that ZO-1 and its upstream interactors VE-cadherin and JAM-A control actomyosin organization and junctional localization of NM2A (Tornavaca et al, 2015). However, in WT bEnd.3 cells we detected only NM2B, but not NM2A, at junctions. This discrepancy could be due to either fixation methods or culture conditions, for example NM2A might redistribute from junctions to cytoplasmic stress fibres when endothelial cells are cultured on a stiff substrate such as glass coverslips. Indeed, how the stiffness of the extracellular matrix affects NM2 distribution, and what is the relative expression and localization of NM2 isoforms in additional types of endothelial cells in vitro and in vivo are interesting questions to address in future studies.

One major goal of this study was to demonstrate the physiological relevance of our observations about the role of cingulin and paracingulin in recruiting NM2s to junctions (Rouaud et al, 2023). Cingulin is concentrated at tight junctions of epithelial cells (Citi et al, 1988; Citi et al, 1989), whereas paracingulin is localized at both tight and adherens junctions (Ohnishi et al, 2004; Guillemot and Citi, 2006; Guillemot et al, 2008; Flinois et al, 2024). There is a substantial body of evidence demonstrating the structural and functional interactions of cingulin and paracingulin with the actomyosin cytoskeleton. Cingulin was discovered as a protein that co-purifies with the actomyosin fraction of intestinal epithelial cells (Citi et al, 1988) and it co-pellets with actin filaments (D’Atri and Citi 2001). Paracingulin associates with actomyosin filaments in cultured cells (Paschoud et al, 2012), and both cingulin and paracingulin have dynamics similar to actin-associated proteins (Paschoud et al, 2011) and interact with GEFs and GAPs that regulate Rho family GTPases (Aijaz et al, 2005; Guillemot et al, 2006b; Guillemot et al, 2008; Elbediwy et al, 2012; Guillemot et al, 2014; Citi et al, 2014). Here we show that in both aortic endothelial cells in vivo and in bEnd3 cells in vitro, paracingulin controls the junctional accumulation of NM2s, whereas cingulin has no role likely because it is expressed at low levels in these cells. Thus, the relevance of cingulin versus paracingulin in the junctional recruitment of NM2s likely depends on their relative levels of expression in specific cell types. Additional studies are required to validate the role of cingulin in recruiting NM2s to junctions of epithelial cells in vivo.

Concerning the mechanisms through which paracingulin regulates NM2s accumulation at endothelial junctions, the direct interaction observed in vitro between purified full-length paracingulin and purified NM2 rod fragments, and the requirement of the NM2-binding rod region of paracingulin to rescue the phenotype of junctional fragmentation in cultured epithelial (Eph4) cells (Rouaud et al 2023) suggest that paracingulin acts by directly binding to NM2s and tethering them to junctions. However, since paracingulin interacts with GEFs and GAPs for Rho GTPases (Guillemot et al, 2008; Elbediwy et al, 2012; Guillemot et al, 2014; Citi et al, 2014), we cannot definitively rule out the possibility that the KO of paracingulin affects NM2 accumulation at junctions, at least in part, by modulating Rho GTPase activation. ZO-1 is expressed ubiquitously and is implicated in the recruitment of both cingulin and paracingulin to junctions (Umeda et al, 2004; Pulimeno et al, 2011; Paschoud et al, 2012; Vasileva et al, 2022; Rouaud et al, 2023), indicating that the loss of junctional NM2A upon ZO-1 depletion (Tornavaca et al, 2015) is an indirect consequence of the decreased accumulation of paracingulin at junctions.

Here we did not investigate the effect of KO of paracingulin on endothelial barrier function, and future studies should address this question. Our previous studies indicate that cingulin and paracingulin are not required for the maintenance of epithelial barriers in confluent monolayers in vitro and in vivo (Guillemot et al, 2004; Guillemot et al, 2012; Mauperin et al, 2023). However, there is evidence for a role of cingulin in the control of endothelial barrier function, since siRNA-mediated depletion of cingulin in primary human pulmonary endothelial cells increases thrombin-induced endothelial cell permeability, associated with increased activation of GEF-H1 and RhoA (Tian et al 2016). Moreover, a role for cingulin in endothelial barrier function in vivo was described in the brain (Schossleitner et al, 2016) and in a burn injury model (Zhuravleva et al, 2020). Thus, we speculate that cingulin expression is heterogeneous within vascular tissues, with a higher expression in a specific subset of endothelial cells, where it plays a role in the control of tight junction barrier function.

A crucial question to be addressed in the future concerns the physio-pathological consequences of the lack of paracingulin at junctions of vascular endothelial cells in vivo. Endothelial cells are exposed to continuous mechanical forces, such as shear stress and cyclic stretch (Fang et al, 2019), and NM2s are key components of the cellular mechano-transduction machinery (Charras and Yap, 2018). In fact, NM2A plays a central role in the stabilization of the endothelial cell cortex during development (Goeckeler et al, 2008; Ma et al 2020), and it was implicated in endothelial sprouting (Fischer et al, 2009), cAMP-mediated endothelial secretion of von Willebrand factor (Li et al, 2018) and maintenance of the blood-brain barrier (Deng et al, 2024). Moreover. actomyosin contractility induced by agonists through activation of myosin light chain kinase weakens endothelial barriers (Shen et al, 2010). However, paracingulin-KO mice are viable and show normal weight and growth (Flinois et al, 2024; Rouaud et al, 2025), suggesting that the loss of paracingulin, and its downstream effects, such as loss of junctional NM2A in endothelial cells, can be compensated for by redundant mechanisms. Thus, perturbation of homeostatic conditions and treatment with agonists is likely required to reveal a role for paracingulin in the regulation of endothelial physiology. In this respect, we recently showed that the contractility and response to agonists of mesenteric arteries and aorta were similar in WT and CGNL1-KO mice (Rouaud et al, 2025), suggesting that junctional paracingulin and NM2s are not critical in the control of endothelium signaling to regulate vascular smooth muscle contractility. The KO of paracingulin also results in altered microtubule organization and epithelial polarity, and loss of the junctional localization of the minus-end microtubule binding protein CAMSAP3 in epithelia in vivo (Flinois et al, 2024). Additional studies are therefore needed to investigate whether paracingulin controls endothelial physiology and barrier function in vivo under baseline conditions or in response to agonists, and the potential mechanistic implications of microtubules and NM2s in the observed phenotypes.

## Methods

Data Mining

Data on mRNA expression levels for *CGN, CGNL1, MYH9* and *MYH10* mRNA (TPM, Transcript Per Million) were obtained by query of the GTEx Portal (https://gtexportal.org/home/multiGeneQueryPage, accessed on 19 February 2025), which collects data from microarray analyses of tissues.

Generation of CGN-KO and CGNL1-KO mice

CGN-KO and CGNL1-KO mice were described previously (Guillemot et al, 2012; Flinois et al, 2024; Rouaud et al, 2025). Mice were euthanized by sodium pentobarbitone administration according to the guidelines of the Swiss Federal Office for Food Security and Veterinary Affairs and the Canton of Geneva and the protocols were approved by the Institutional Animal Care Committee of Animal Care of the Canton of Geneva (Animal Experimentation Permits n. GE/1027/3853/3, GE/31.1.1010/1800/I, GE/67/15, GE/9/18, GE/68/17, GE/133/20 to SC).

Cell culture experimental model

The mouse brain microvascular endothelial (endothelioma) cell line (bEnd.3) was cultured at 37°C, 5% CO2 in Dulbecco’s Modified Eagle’s Medium (DMEM) containing 10% of fetal bovine serum (FBS) and 1% non-essential amino acids (Vasileva et al, 2017).

Cell clones KO for CGNL1 were generated using CRISPR–Cas9 gene editing technology as described for mCCD CGNL1 KO cells (Vasileva et al 2022). The Extended Data Figure shows validation of deletion of CGNL1 by CRISPR/Cas9 in WT bEnd.3 background by genomic sequencing (A) and immunoblotting analysis (B). Immunofluorescence data are shown for clone 10 and were reproduced in the second clone.

Antibodies

The following primary antibodies against the indicated proteins were used for immunofluorescence microscopy (IF) and immunoblotting (IB) at the indicated dilutions: mouse anti-cingulin (in house monoclonal 22BD5A1; IF: 1:500); rabbit anti-CGNL1 (in house, 20893, IF/IB: 1/1000); mouse anti-ZO-1 (Thermofisher 33-9100, IF: 1/1000); rabbit anti-NM2A (BioLegend 909801, IF: 1/300, IB: 1/1000); rabbit anti-NM2B (BioLegend 909901, IF: 1/300, IB: 1/1000); goat anti- VE-Cadherin (sc6458, IF: 1/500), mouse anti-beta-tubulin (Thermofisher 32-2600, IB: 1/1000).

Secondary antibodies for immunofluorescence from Jackson ImmunoResearch were diluted 1/300: and anti-goat-IgG (705-546-147) conjugated to Alexa Fluor 488; anti-rabbit-IgG (711-165-152) conjugated to Cy3; anti-mouse-IgG (115-605-174) conjugated to Alexa Fluor 488; anti-rat-IgG (712-175-153) conjugated to Cy5.

Immunofluorescence

Thoracic-abdominal aorta tissues were fixed in 4% formaldehyde and processed for immunofluorescence microscopy as described in (Rouaud et al, 2025). Immunofluorescence labeling of bEnd.3 cells was carried out using the protocols described in (Rouaud et al, 2023) for cultured epithelial cells..

Slides were imaged on a Zeiss LSM800 confocal microscope using a Plan-Apochromat 63×/1.40 oil objective (1,024×1,024 px). Maximum intensity projections of z-stack images (typically 3–6 confocal planes over 1.0–1.5 μm, step size = 0.3–0.6 μm) for aorta and bEnd.3 were obtained. Images were extracted from czi file using ImageJ, adjusted and cropped using Adobe Photoshop, and assembled in Adobe Illustrator for figures.

## Data Availability

Description: Extended Data Figure Legend: A) Validation of Crispr/Cas9-mediated deletion of CGNL1 in two distinct clonal lines of bEnd.3 cells by genomic sequencing. The WT sequence is shown on top. B) Validation of KO by immunoblotting analysis of the expression of CGNL1, NM2A, NM2B and ZO-1 in WT and CGNL1-KO bEnd.3 cells. Beta-tubulin was used as a loading control. Numbers on the left refer to migration of molecular weight standards.. Resource Type: Image. DOI: https://doi.org/10.22002/mp0ys-6b272
